# Photophysical Characteristics of Multicolor Emitting MDMO-PPV–DMP/ZnO Hybrid Nanocomposites

**DOI:** 10.3390/molecules27030843

**Published:** 2022-01-27

**Authors:** Bandar Ali Al-Asbahi, Arwa Alhamedi Alanezi, Mohamad S. AlSalhi

**Affiliations:** Department of Physics & Astronomy, College of Sciences, King Saud University, P.O. Box 2455, Riyadh 11451, Saudi Arabia; 439203076@student.ksu.edu.sa (A.A.A.); malsalhi@ksu.edu.sa (M.S.A.)

**Keywords:** hybrid nanocomposite, photophysical properties, MDMO-PPV–DMP, ZnO NPs

## Abstract

The tuning of photophysical properties of the poly[2-methoxy-5-(3,7-dimethyl-octyloxy)-1,4-phenylenevinylene]—end capped with dimethylphenyl (DMP), MDMO-PPV–DMP, was achieved by incorporation of ZnO NPs with various contents. Hybrid nanocomposites of MDMO-PPV–DMP with ZnO NPs were prepared by solution blending method and then deposited onto glass substrates. The structural properties of the hybrid nanocomposites samples were characterized using X-ray diffraction, FTIR, and FE-SEM, while their optical properties were extracted from the absorption and photoluminescence spectra. The energy band gap, energy tail, steepness parameter, and CIE chromatic coordinates were tuned by increase the content of ZnO NPs into the polymer matrix. The ZnO NPs incorporation assists the emission wavelength shift and multicolor emitting from the hybrid nanocomposites.

## 1. Introduction

Numerous types of electroluminescent and conducting polymers have been specified as a promising kind of materials for optoelectronic applications such as organic light emitting diodes (OLEDs), organic photovoltaic cells, organic phototransistors, and organic photo detectors. New research based on the conductive polymers has been opened up to improve a new technology of plastic electronics. When the conducting polymers are forward biased by direct current, they emit light via holes–electrons recombination. The energy difference between the lowest unoccupied molecular orbit (LUMO) level and highest occupied molecular orbit (HOMO) level will play a crucial role in determining the light color (emitted photon wavelength).

The manufacture of the optoelectronic devices based on derivatives of poly (p-phenylene vinylene) (PPV) has attracted the interest of researchers. In particular, poly[2-methoxy-5-(3,7-dimethyl-octyloxy)-1,4-phenylenevinylene], MDMO-PPV, is one of the most broadly considered of these derivatives because of its high stability, unique electroluminescent properties, and particular structure that allow easy deposition in flexible circuits, large-area electronics, and low-cost printable electronics [1,2,3,4]. Moreover, MDMO-PPV is an electron donating material and has acquired historical importance as the material for which electroluminescent in conjugated polymers was discovered in 1990 [5].

According to numerous studies it was found that the OLED devices based on polymers inherit two major problems, namely, poor optical absorption and lack of carrier mobilities [6]. To overcome the major problems of the OLEDs and thus improve their performance, the inorganic nanostructures were incorporated into the polymer [7,8]. Numerous studies have been reported on optoelectronic devices based on nanocomposite of polymer and metal oxide nanoparticles. It was found that the nanocomposites improve the stability and the optoelectronic properties of the organic devices. Despite this, the optical and structural properties of the polymer are still a topic of investigation. Baraton and co-workers reported that the conjugation lengths of PPV do not break upon inclusion of TiO2 NPs [9]. However, Yang and co-workers observed that the PPV conjugation length was gradually reduced with increments of the SiO_2_ NPs [10].

In this paper, ZnO NPs successfully dispersed into the poly[2-methoxy-5-(3,7-dimethyl-octyloxy)-1,4-phenylenevinylene]—end capped with dimethylphenyl (MDMO-PPV–DMP) for better photophysical properties and tuning of color emission. ZnO NPs is an n-type semiconducting material with a wide bandgap, high exiton binding energy, high optical transparency, and is nontoxic. MDMO-PPV is p-type polymer, light-emitting polymer, soluble in common organic solvents, and widely used in photovoltage devices. Thus, tailoring the property of both ZnO and polymer, and creating a nanocomposite hybrid with enhanced properties can be achieved by incorporation of ZnO NPs with emitting polymers. For this purpose, the influence of the incorporation of various weight percentages of ZnO NPs on the photophysical properties of the MDMO-PPV–DMP/ZnO hybrid nanocomposite will be explained in detail.

## 2. Results and Discussion

**Figure 1:**
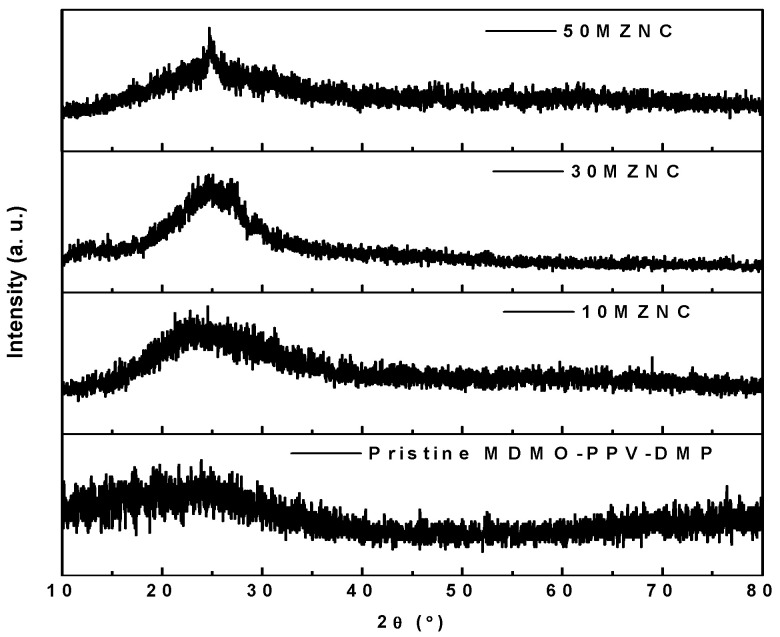
XRD diffractograms of pristine MDMO-PPV–DMP and MZNCs thin films.

The X-ray diffractograms of the thin films of pristine MDMO-PPV–DMP, and MZNCs are shown in Figure 1. The broad band in the 2θ range of 15–30° confirms the amorphous phase of the pristine MDMO-PPV–DMP. The sample’s crystallinity was slightly enhanced upon incorporation of the ZnO NPs as evidenced from the narrowing the broad band of the MDMO-PPV–DMP. The broad band became clearer and less broadening with the increase of the ZnO NPs content, confirming the incorporation of ZnO NPs into the MDMO-PPV–DMP matrix and the nanocomposite structure formation [11]. Moreover, the broad band of MDMO-PPV–DMP was hardly observed in the diffractograms of the hybrid 50MZNCs due to lower scattering factors compared to ZnO NPs.

**Figure 2:**
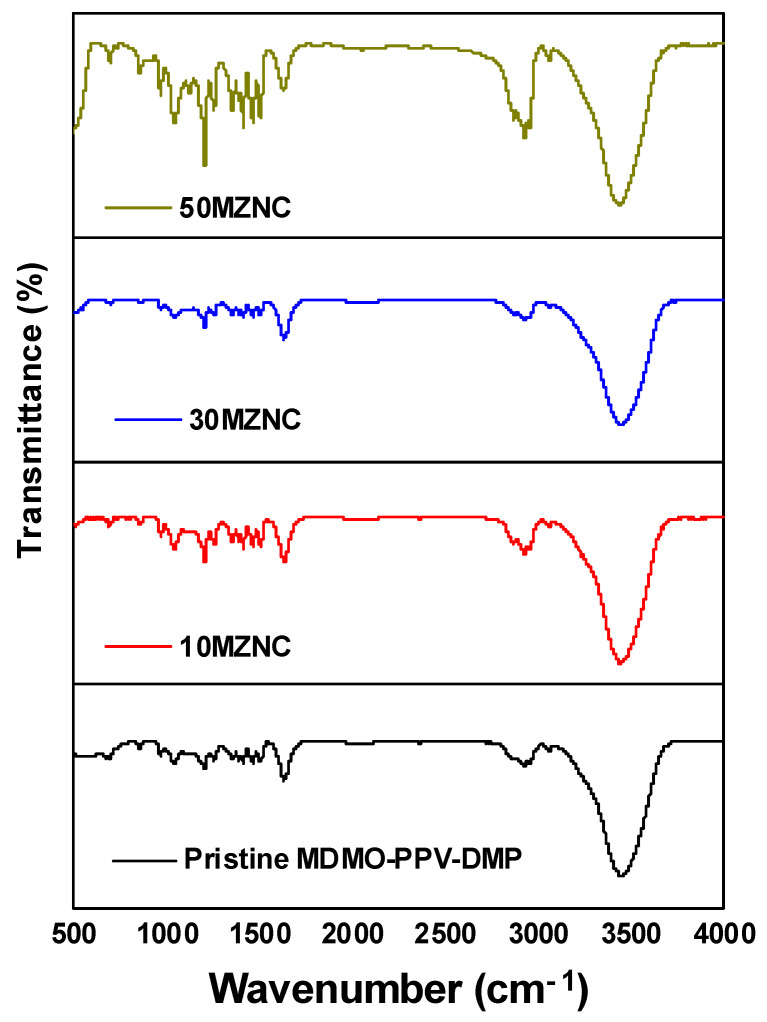
FTIR spectra of pristine MDMO-PPV–DMP and the MZNCs thin films.

The nature of the chemical groups of pristine MDMO-PPV–DMP and the hybrids of MZNCs can be seen in the FTIR spectra in Figure 2. Many bands in the range of 4000–400 cm^−1^ were revealed for all samples. The transmission peaks at 3061 and 3450 cm^−1^ assigned to C-H stretching in the benzene ring and O-H stretching, respectively, whereas the vibrational modes at 2926 cm^−1^, 2951 cm^−1^, and 2860 cm^−1^ denote the asymmetrical and symmetrical CH_2_ and CH_3_ stretching. The preparation of the samples disks in an open-air atmosphere resulted in formation band at 1633 cm^−1^ due to H-O-H bending vibration mode [12]. Two semicircular stretch modes can be assigned to the phenyl ring at 1506 and 1413 cm^−1^ [13]. The bands at 1472 and 1354 cm^−1^ refer to antisymmetric and symmetric alkyl CH_2_, respectively. The peaks at 1252, 1202, and 1040 cm^−1^ are associated with C-O stretching in ether groups, phenyl oxygen stretch, and alkyl oxygen stretch, respectively [11,14]. The bands at 957 cm^−1^ and 855 cm^−1^ are associating with trans double bond vinylene C-H wagging and phenyl C-H wagging, respectively, which indicates the dipole normal to the phenyl vinyl plane [15]. A new band at 1092 cm^−1^ was detected in the transmission spectra of MZNCs with a gradual increase up to 1126 cm^−1^ upon incorporation of ZnO NPs. This shifting in the new band can be attributed to the deprotonating that signifies the transfer or removal of protons (H^+^ ions) due to O^2−^ in ZnO and thus induces stress in the C-H bonding [16].

**Figure 3:**
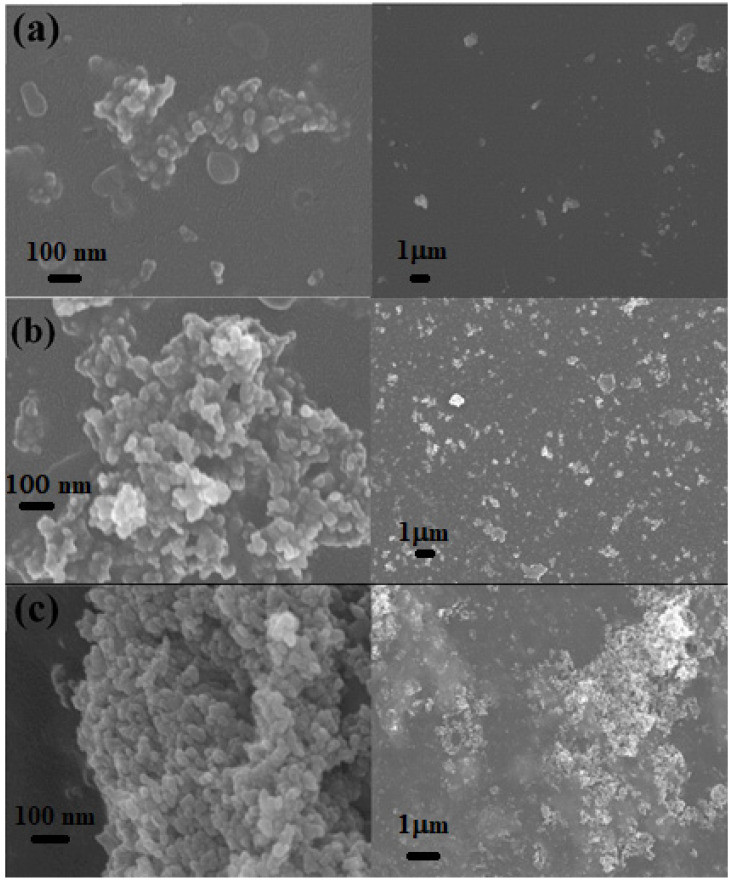
FE-SEM images of (**a**) 10MZNC, (**b**) 30MZNC, and (**c**) 50MZNC thin films under scales of 100 nm and 1μm for each thin film.

Figure 3 displayed the surface morphology of MZNCs thin films under scales 100 nm and 1μm. The distribution of the ZnO NPs on the MDMO-PPV–DMP matrix can be clearly observed. The images with a scale of 100 nm confirmed the uniform particle shape of the ZnO NPs, while the homogeneous distribution of the nanoparticles on the matrix thin film with presence agglomeration at the high content of ZnO NPs (≥30 wt.%) was observed from the images with a scale of 1μm. This agglomeration with inclusion of ZnO NPs in the MDMO-PPV–DMP matrix plays crucial role to enhance the conformational disorder [17,18,19].

**Figure 4:**
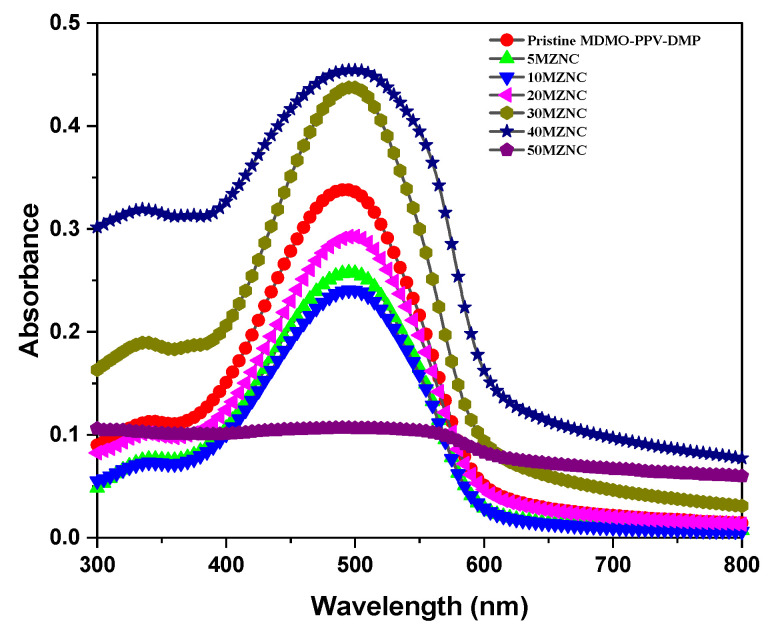
Absorption spectra of pristine MDMO-PPV–DMP and the MZNCs thin films.

The absorption spectra of all thin films of pristine MDMO-PPV–DMP and MZNCs were demonstrated in Figure 4. Two main absorption peaks were detected for pristine MDMO-PPV–DMP at 342 and 492 nm, corresponding to 0–1 and 0–0 vibronic transitions, respectively. The increase of the absorbance can be ascribed to scattering caused by the ZnO NPs in the MDMO-PPV–DMP matrix, which is expected to rise in increments with the ZnO NPs content. The main absorbance peak at 492 was slightly red shifted with increments with the ZnO NPs content and reached to 499 nm for 40MZNC. This result may lead to the possibility of extending conjugation length of the MDMO-PPV–DMP when the ZnO NPs content is increased [20]. The non-uniform change in the absorbance with the ZnO NPs content is due to the change in the homogeneous distribution quality with increments with the ZnO NPs content. At a low content of ZnO NPs (5 and 10 wt.%), the absorbance decreased due to good distribution into the polymer matrix and then dramatically increased with increments with the ZnO NPs content (20–40 wt.%). This rise in absorbance could be justified in terms of increasing the aggregation with increasing ZnO NPs content. This is unlike the behavior which occurred with 50MZNC, where the absorption became nearly constant for the entire wavelength range, indicating aggregation of the MDMO-PPV–DMP chain or as a result of the relatively long conjugated chains. Similar behavior was observed with another system of MEH-PPV/CdTe nanocomposite at a high content of the CdTe nanoparticles [21].

**Figure 5:**
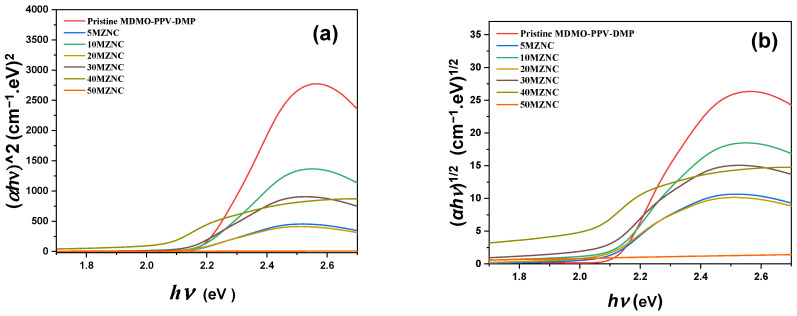
(**a**) plot of (*αhν*)^2^ vis. *hν*, and (**b**) (*αhν*)^1/2^ vis. *hν* plot for pristine MDMO-PPV–DMP and the MZNCs thin films.

The influence of the ZnO NPs content on the values of direct and indirect energy band gap (*E*_gd_ and *E*_gi_) of the MDMO-PPV–DMP thin film could be estimated by the extrapolating method using the (*αhν*)^2^ and (*αhν*)^1/2^ versus photon energy (*hν*) plot, respectively, as presented in Figure 5, where *α* is the absorption coefficient. The values of *E*_gd_ and *E*_gi_ can be obtained by extrapolation of the linear portion of each curve in Figure 5, and then listed in Table 1. The *E*_gd_ and *E*_gi_ values of MDMO-PPV–DMP thin film were found to be 2.18 eV and 2.06 eV, respectively. These obtained band gaps of MDMO-PPV–DMP are in agreement with previous reports [22,23]. These values of both *E*_gd_ and *E*_gi_ were dramatically reduced upon increment of ZnO NPs to reach 2.03 eV and 1.89 eV, respectively. The reduction in the energy band gap of the MDMO-PPV–DMP indicates that the conduction band and the valence band are getting closer to each other. The presence of both types of energy band gaps in MZNCs thin films can broaden the range of absorption energy to improve the photoelectric energy conversion [24]. Moreover, the close values of both *E*_gd_ and *E*_gi_ from each other make such that these hybrids are an effective material in optoelectronic devices.

**Figure 6:**
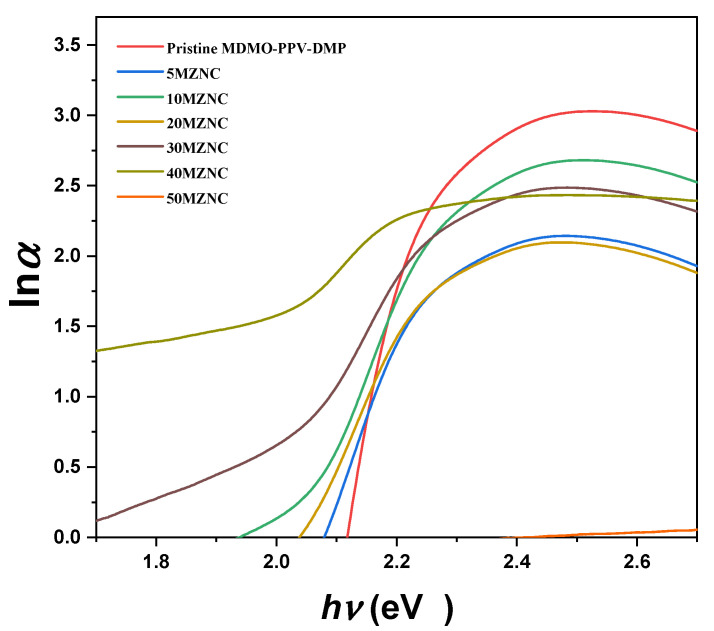
Urbach plots of pristine MDMO-PPV–DMP and the MZNCs thin films.

The tail width of localized states in the forbidden band gap can be reflected by energy gap tail (*E*_u_), which is less than the band gap energy. The Urbach plots for all thin films are displayed in Figure 6. The *E*_u_ values were estimated from the inverse slope of the linear part of the Urbach plots [25], as seen in Table 1. The higher incorporation of the ZnO NPs into the MDMO-PPV–DMP resulted in a systematic increase in the *E*_u_ values and thus formation of imperfections and disorder in the band structure of the nanocomposites in addition to the creation of more localized states within the forbidden gap [26,27]. The shrinkage/widening of the optical absorption edge can be described by the steepness parameter ($\sigma =K_{\beta }T/E_{u}$), where *T* and *k*_β_ are the absolute temperature and the Boltzmann constant, respectively. As demonstrated in Table 1, the σ value was decreased with increment of the ZnO NPs. This decrease can be ascribed to the rise in the localized density of electronic states within the forbidden band gap of the hybrid nanocomposites [18,28].

**Figure 7:**
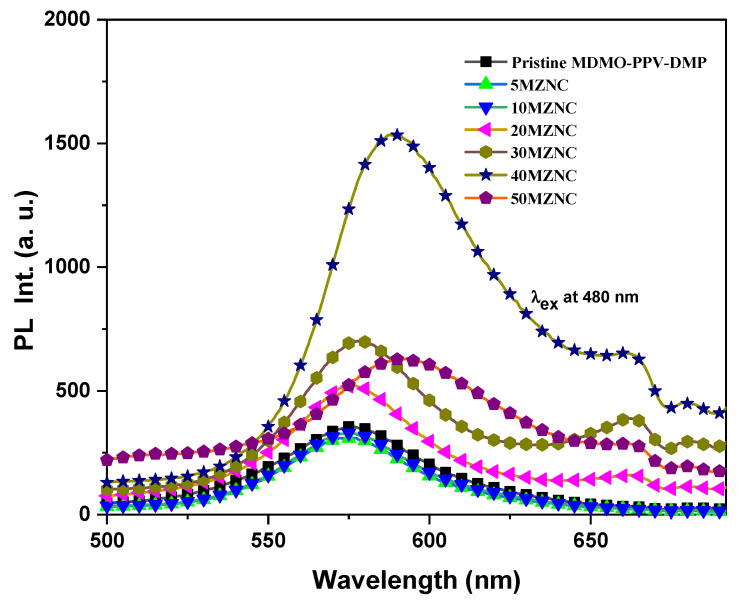
PL spectra of pristine MDMO-PPV–DMP and the MZNCs thin films.

The photoluminescence (PL) spectra were recorded in Figure 7 to study the behavior of the molecules in the excited state in the absence and presence of the ZnO NPs with various ratios. The PL spectra were collected for wavelengths in the range of 500 to 700 nm with the excitation wavelength fixed at 480 nm. At low content of ZnO NPs (5 and 10 wt.%), no significant change was observed. At higher content, significant changes in both PL intensity and shifting of the PL peak were detected. The emission intensity of the MDMO-PPV–DMP with the main peak at 575 nm was systematically enhanced upon increment the ZnO NPs and red shifted to reach 592 nm at 50 wt.%. The improvement in the intensity can be interpreted to chain separation and/or the charge trapping effect [18,29]. As ZnO is an n-type semiconductor with a stronger electron affinity, more holes can be allowed to recombine through the MDMO-PPV–DMP/ZnO interface and thus enhance the emission intensities [30]. The maximum emission was detected for 40MZNC. The quenching in the emission intensity by incorporation more ZnO NPs (50 wt.%) can be attributed to that the higher aggregation resulted in plenty of electrons which were mostly blocked around the ZnO surface and confined the recombination population for the nanocomposite hybrid of MDMO-PPV–DMP/ZnO. Similar behavior was observed with other systems such as PFO-oxe/ZnO [29]. On the other hand, the red shift in the PL spectrum is associated with changes in the conjugation length [31]. The inclusion of the ZnO NPs in the MDMO-PPV–DMP matrix leads to separation in the polymer chains and thus changes in the conjugation length of the polymer resulting in a red shift in the PL spectra. Moreover, a new peak at 663 nm can be noted at high content of ZnO NPs (≥20 wt.%), which can be attributed to intrachain electronic states [32].

**Figure 8:**
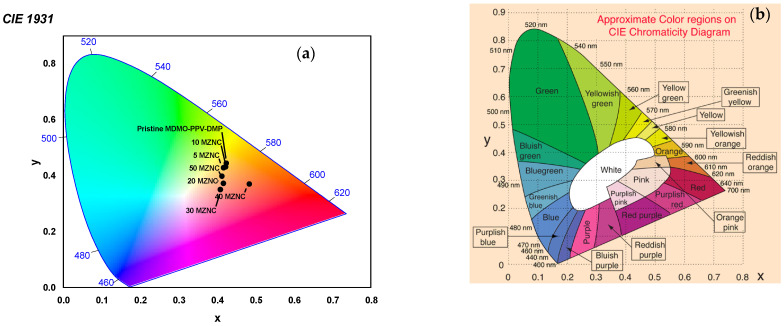
(**a**) (x, y) chromaticity coordinates generated from pristine MDMO-PPV–DMP and the MZNCs thin films, (**b**) CIE 1931 color space chromaticity diagram.

Figure 8a shows the CIE chromaticity diagram which identifies the perceived color of the emission spectrum of pristine MDMO-PPV–DMP and the MZNCs thin films. The CIE 1931 chromaticity diagrammed in Figure 8b was used as a reference [33,34] to characterize the color emission generated from each thin film that was excited at 480 nm. The (x, y) coordinates corresponding to all thin films are presented in Table 2. As the ZnO NPs contents are increased, the (x, y) coordinates are turned from the greenish yellow region to the white region on the chromaticity diagram. It can be concluded that the new peak that is created in the PL spectra (Figure 7) at ≥20 wt.% of ZnO NPs played a crucial role to produce the white emission.

## 3. Materials and Methods

MDMO-PPV–DMP, with a molecular weight of 100,000 g/mol was purchased from American Dye Source, Inc. (Morgan Boulevard, QC, Canada), and ZnO NPs with a mean size of ~25 nm in wurtzite phase, was purchased from Sigma-Aldrich (St. Louis, MO, USA).

The nanoparticles of ZnO were dispersed into ethanol with 2 vol.% of Ethylene Glycol, stirred at 600 rpm for 2 h, and sonicated for 1 h to obtain a homogeneous solution. By the solution blending method, ZnO NPs with different ratios (5, 10, 20, 30, 40, and 50 wt.%) were incorporated into a fixed toluene solution of MDMO-PPV–DMP (5 mg/mL). The hybrid of MDMO-PPV–DMP with 5, 10, 20, 30, 40, and 50 wt.% of ZnO NPs were labeled as 5MZNC, 10MZNC, 20MZNC, 30MZNC, 40MZNC, and 50MZNC, respectively. Fifty milliliters of each solution of pristine MDMO-PPV–DMP and MZNCs were deposited into cleaned glass substrates by spin coating technique (2000 rpm, 30 s) to prepare homogeneous thin films. All the thin films were dried at 120 °C for 10 min at ambient conditions prior to carry out all measurements.

X-ray diffraction (XRD; Miniflex 600, Rigaku, Latvija, Japan), Fourier transform infrared spectroscopy (FT-IR; PerkinElmer Spectrum 100, Waltham, MA, USA), and field emission scanning electron microscope (FE-SEM; JEOL JSM-7610F Tokyo, Japan) were utilized to characterize the structural properties of the hybrid nanocomposites. The optical properties of the samples were investigated by UV–vis Spectrophotometer (JASCO V-670, Cremella, Italia) and spectrofluorometer (JASCO FP-8200, Cremella, Italia). Moreover, the OriginLab program version 2019b (Northampton, MA, USA) was employed to obtain the CIE coordinates of all thin films from their emission spectra.

## 4. Conclusions

The incorporation of ZnO NPs with various content was successfully effective on both the optical and structure properties of MDMO-PPV-DMP. The crystallinity of the samples was slightly enhanced upon incorporation of the ZnO NPs. The homogeneous distribution of the ZnO NPs on the MDMO-PPV–DMP matrix was confirmed by FE-SEM images. The conjugation length of the MDMO-PPV–DMP was extended when the ZnO NPs content was increased. Both direct and indirect types of energy band gaps were found in MZNCs thin films and dramatically reduced upon increment of the ZnO NPs content. The formation of imperfections and disorder in the band structure of the nanocomposites in addition to the creation more localized states within the forbidden gap can be expected from a systematic increase in the Eu values and reduction in the s values upon incorporation of ZnO NPs into the MDMO-PPV–DMP. The inclusion of the ZnO NPs in the MDMO-PPV–DMP matrix leads to enhancement of the PL intensity and tuning of the color emission. A new emission peak at 663 nm was noted at a high content of ZnO NPs (≥20 wt.%), which can be attributed to intrachain electronic states, and played a crucial role to produce the white emission. The results obtained in this work confirm that the hybrid of MZNCs is an effective material if used in optoelectronic devices.

## Figures and Tables

**Table 1:** Optical parameters of pristine MDMO-PPV–DMP and the MZNCs thin films.

Specimen	Egd (eV)	Egi (eV)	Eu (eV)	$\sigma =K_{\beta }T/E_{u}$
Pristine MDMO-PPV–DMP	2.18	2.06	0.0418	0.6143
5MZNC	2.14	2.05	0.0888	0.2892
10MZNC	2.16	2.01	0.0950	0.2705
20MZNC	2.14	2.03	0.1029	0.2496
30MZNC	2.11	2.04	0.1303	0.1971
40MZNC	2.03	1.89	0.2238	0.1147
50MZNC	-	-	0.2012	0.1277

**Table 2:** Experimental CIE coordinates corresponding of pristine MDMO-PPV–DMP and the MZNCs thin films.

Specimen	X	Y	Color
Pristine MDMO-PPV-DMP	0.4238	0.4446	Greenish yellow
5MZNC	0.4167	0.4283	Greenish yellow
10MZNC	0.4221	0.4316	Greenish yellow
20MZNC	0.4126	0.3980	white
30MZNC	0.4083	0.3501	white
40MZNC	0.4838	0.3700	Orange pink
50MZNC	0.4165	0.3724	white

## Data Availability

Not applicable.

## Sample Availability

Samples of the compounds MZNCs are available from the authors.
